# Predicting Absolute Risk of First Relapse in Classical Hodgkin Lymphoma by Incorporating Contemporary Treatment Effects

**DOI:** 10.3390/cancers17172760

**Published:** 2025-08-24

**Authors:** Shahin Roshani, Flora E. van Leeuwen, Sara Rossetti, Michael Hauptmann, Otto Visser, Josée M. Zijlstra, Martin Hutchings, Michael Schaapveld, Berthe M. P. Aleman

**Affiliations:** 1 Department of Epidemiology, Netherlands Cancer Institute, Plesmanlaan 121, 1066 CX Amsterdam, The Netherlands; s.roshani@nki.nl (S.R.);; 2 Department of Hematology, Copenhagen University Hospital, Rigshospitalet, 2100 Copenhagen, Denmark; 3 Institute of Biostatistics and Registry Research, Brandenburg Medical School Theodor Fontane, 16816 Neuruppin, Germany; 4 Department of Registration, The Netherlands Comprehensive Cancer Organisation, Boven Clarenburg 2, 3511 CV Utrecht, The Netherlands; 5 Department of Hematology, Amsterdam University Medical Center, Location VUmc, Cancer Center Amsterdam, De Boelelaan 1117, 1081 HV Amsterdam, The Netherlands; 6 Department of Clinical Medicine, Faculty of Health Sciences, University of Copenhagen, 2200 Copenhagen N, Denmark; 7 Department of Radiation Oncology, Netherlands Cancer Institute, Plesmanlaan 121, 1066 CX Amsterdam, The Netherlands

**Keywords:** Hodgkin lymphoma, relapse, absolute risk, prediction, validation

## Abstract

**Background/Objectives**: There is a need for prediction models which enable weighing benefits against risks of different treatment strategies for individual Hodgkin lymphoma (HL) patients. Therefore, we aimed to predict absolute risk of progression, first relapse or death (PRD) with and without incorporating HL treatment. **Methods**: The prognostic and treatment information of 2343 patients treated for classical HL at ages 15–60 years between 2008 and 2018 in the Netherlands was used to predict absolute risk of PRD up to 5 years after diagnosis using Cox proportional hazard models allowing for time-varying coefficients. Models were externally validated in 1675 patients treated for classical HL in Denmark between 2000 and 2018. **Results:** In early stages, gender, leukocyte, and lymphocyte counts were associated with risk of PRD. Additionally, receiving >4 cycles of ABVD (doxorubicin, bleomycin, vinblastine, and dacarbazine) or ABVD plus radiotherapy predicted lower risk of relapse compared with receiving ≤4 cycles of ABVD. In advanced stages, age, albumin and leukocyte counts predicted PRD risk. Receiving (escalated) BEACOPP (bleomycin, etoposide, doxorubicin, cyclophosphamide, vincristine, procarbazine, prednisone) predicted lower PRD risk compared to ABVD. In Danish patients treated between 2008 and 2018, adding treatment information improved 5-year Inverse Probability of Censoring Weighted (IPCW) Area Under the Curve (AUC) values from 0.63 (95% Confidence Interval (CI): 0.55–0.72) to 0.71 (95% CI: 0.63–0.79) in early stages (*p*-value = 0.04) and from 0.59 (95% CI: 0.52–0.65) to 0.62 (95% CI: 0.55–0.68) in advanced stages (*p*-value = 0.33). **Conclusions**: We developed well calibrated models with reasonable discrimination, not only incorporating pre-treatment prognostic factors but also treatment effect enabling the prediction of absolute risk of first relapse/progression.

## Simple Summary

Hodgkin lymphoma (HL) patients have a good prognosis but are at increased risk of various long-term treatment-related complications. It is important to individualize HL treatments in order to prevent relapse and to minimize the risk of long-term complications that might arise due to treatment. For this purpose, we need risk estimators for relapse and for long-term complications. In this study we created models that are able to estimate absolute risk of relapse, based on the clinical presentation of the patient (i.e., age, sex, extent of disease and blood-based markers) as well as proposed treatment strategy, using the information of 2343 classical HL patients treated in the Netherlands between 2008 and 2018. We validated our models on HL patients treated between 2000 and 2018 in Denmark. The results showed that our models accurately predicted relapse risk and were good at telling apart patients who did relapse from those who did not.

## 1. Introduction

Hodgkin lymphoma (HL) is a highly curable malignancy with survival rates currently exceeding 80% at 5 years after diagnosis [1,2]. However, many studies have shown that HL treatment may come at a price, as HL survivors experience increased risks of second malignancies, cardiovascular disease, and other complications [3,4,5,6,7]. Given the high cure rate, optimizing predictive models to weigh benefits against risks of different treatment strategies for individual patients is of paramount importance. Several prognostic models have previously been developed to identify HL patients at higher risks of relapse, including models for early stage patients from the German Hodgkin Study Group (GHSG) [8] and the European Organization for Research and Treatment of Cancer (EORTC) [8] and, for advanced stage patients, the International Prognostic Score (IPS) [9] and the recently published Advanced-Stage Hodgkin lymphoma International Prognostic Index (A-HIPI) [10] model, developed by the HoLISTIC consortium. None of these models include treatment information. Inclusion of treatment information is, however, needed to allow weighing the relapse risk versus the risks of late complications of specific treatments. In addition, adding treatment information to models can create treatment confirmation tools for the current era of HL treatment and can also help reveal treatment effects that are adjusted for important pre-treatment clinical factors [11]. Given the recent advances in treatment, it is crucial to develop and validate prognostic models based on data from more contemporary patients. Therefore, the goal of our project is not only to provide a new prognostic model based on more recently treated patients, but also to investigate the possible predictive information provided by adding a treatment to prognostic models.

## 2. Materials and Methods

### 2.1. Study Population

For this study over the years 2021 and 2022, the population-based Netherlands Cancer Registry actively abstracted and provided detailed clinical characteristics and treatment information for all patients treated for HL in the Netherlands at ages of 15 to 60 years between 2008 and 2010 and 2014–2018. Data on relapse after HL treatment was collected up to January 2024 to have at least 5 years of follow-up for all patients. Patients treated in 2008–2010, although representing somewhat older treatment regimes, were specifically included to also allow the prediction of 10-year relapse risk and mortality. Patients treated between 2011 and 2013 were not included to limit the costs of data collection. Patients with nodular lymphocyte-predominant HL (morphology code: M9659/3, *n* = 231) were excluded, leaving 2343 patients with classical HL for analysis. Of these, 850 (36.3%) were diagnosed between 2008 and 2010, and 1493 (63.7%) between 2014 and 2018. For all patients, information was available on age, concentrations of hemoglobin, albumin, leukocytes, and lymphocytes in the blood as continuous variables plus gender, presence of extra-nodal disease, Ann-Arbor stage, number of affected lymph node stations, presence of B-symptoms, and LDH (normal/abnormal) as categorical predictors. The number of affected extra-nodal localizations was not available for patients diagnosed between 2008 and 2010. For patients with Ann Arbor stage 4 HL in this period, we assumed the presence of at least one extra-nodal localization.

Most patients were treated according to the standard arms of stage specific trial protocols of the EORTC or the GHSG applied at the time. Available treatment information included chemotherapy regimens and number of cycles, radiotherapy location and principle (involved node, involved field, other, and unknown), total radiation dose and number of fractions. An important clinical dilemma for early stages (Ann Arbor stage I/II) is whether to add radiotherapy to chemotherapy, typically consisting of doxorubicin, bleomycin, vinblastine, and dacarbazine (ABVD), while for advanced stages (Ann Arbor stage III/IV) the dilemma is whether to use ABVD or an (escalated) bleomycin, etoposide, doxorubicin, cyclophosphamide, vincristine, procarbazine, prednisone (BEACOPP) or a BEACOPP-like regimen. Therefore, we created stage-specific treatment variables to address the possible benefit of adding radiotherapy for early stage patients and more intensive chemotherapy for advanced stage patients (see Supplement S1 for further details).

This study was declared outside the scope of the Medical Research Involving Human Subject Act by the Institutional Review Board of the Netherlands Cancer Institute (IRBd20-332, dated 4 December 2020, and IRBd25-109, dated 17 April 2025) and the need for individual informed consent was waived, as existing data from medical files and (cancer) registries were used.

### 2.2. Statistical Analysis

Time to progression, relapse or death due to all causes (PRD) defined as progression-free survival (PFS) and time to death due to all causes defined as overall survival (OS) were assessed in days since HL diagnosis. Follow-up information was complete until January 2024. For modeling PFS in the current study, all patients were censored after 5 years (1825 days). Patients with missing stage information were excluded from the analysis (3 patients of whom 2 patients experienced PRD). The Cox proportional hazards (PH) model was used to predict risk of PRD for each individual. Details of the modeling steps are given in Supplement S2. In case a pair of variables had a >0.5 association strength, one of these variables was removed from the initial variables set (Supplements S3). Among advanced stage patients, hemoglobin was strongly associated with albumin (association strength > 0.5), while the presence of extra-nodal disease was strongly associated with stage. Hemoglobin and the presence of extra-nodal disease were subsequently removed from the initial variable set (Supplemental Figure S3). Functional forms of continuous predictors were evaluated through locally estimated scatterplot smoothing (LOESS) curves of their values against martingale residuals of the null Cox model [12] and in case of non-linearity, piecewise linear forms that best matched the LOESS curve were created (Supplement S4). Missing values were addressed by multiple imputation with 10 iterations using random forest as its internal predictive algorithm. To avoid overfitting and to derive one unique model to predict risk for different time points, the *p*-value based backward elimination of included terms was used based on the D2 statistic [13] of nested model comparisons from imputed sets. The PH assumption was checked by pooling chi-square statistics of independence tests between Schoenfeld residuals and time [14] over imputed datasets resulting in a D2 statistic with an approximate F distribution [13]. Time-varying coefficient(s) with the form of step functions of time were created for variable(s) violating the PH assumption [15]. The number and the location of cut-points in time were chosen based on the data and guided by LOESS curves of Schoenfeld residuals against time [14] in order to obtain proportional hazards in each time interval. Danish HL patients diagnosed between 2000 and 2018 were used for the validation of all models but, since our models were developed based on Dutch patients diagnosed between 2008 and 2018, we also validated PFS models in the subset of Danish patients diagnosed between 2008 and 2018. We used Inverse Probability of Censoring Weighted (IPCW) Area Under the Curve (AUC) [11,16] over absolute risk estimates of individuals as a loss function. Random forest imputation (by mice package [17]) was used to impute missing values among Danish HL patients in early or advanced stages. Differences between external IPCW AUC values of prognostic and treatment-based models were compared with the Delong test [18]. We also compared the 5-year IPCW AUC of our advanced stage models with IPS and A-HIPI. Details on how the absolute risks were estimated are provided in Supplement S5. Quantile-based calibration plots were made by dividing the risk estimates into 5 bins. All analyses were performed using R (version: 4.4.1) and RStudio (version: 2024.09.0). Information on the packages used can be found in Supplement S6. The full code including written functions can be found at [https://github.com/Shahin-Roshani/cHL.relapse.codes_for_cancers.paper/blob/main/cHL_relapse_codes.R]. Our developed models for PRD are available as easy to use online risk calculators at [https://shahin-roshani.shinyapps.io/cHL_relapse_app/].

## 3. Results

In total, 1337 (57.1%) classical HL patients had early-stage disease and 1003 (42.1%) had advanced-stage disease. Median follow-up time was 7.8 years (Inter Quartile Range (IQR) = 5.3 years, Table 1). In total, 331 patients progressed, had a relapse or died within 5 years of their diagnosis of whom 132 patients had early stage (Ann Arbor stage I/II) disease, 197 had advanced stage disease (Ann Arbor stage III/IV), and 2 patients had missing stage information. The 5-year cumulative incidence of PRD was 9.9% (95% Confidence Interval (CI): 8.4–12.0%) among early-stage and 20.0% (95% CI: 17.0–22.0%) among advanced-stage patients, respectively. During follow-up, 148 patients died, translating into a 5-year cumulative mortality of 3.9% (95% CI: 3.2–4.8%); 2.3% (95% CI: 1.6–3.2%) in early stage and 6.0% (95% CI 4.7–7.6%) in advanced stage. A total of 14 out of 30 (47%) early-stage patients and 34 out of 61 (56%) advanced-stage patients who died within the first 5 years, experienced relapse or progression prior to the date of death. Of 30 early-stage and 61 advanced-stage patients who died within 5 years, 5 (17%) and 16 (26%) died within the first year of HL diagnosis without prior record of progression or relapse, respectively.

### 3.1. Models for Progression-Free Survival

In early stages, male sex and higher leukocyte counts were associated with higher risk of PRD, but the effect of leukocyte count lessened greatly for values ≥12 × 10^9^/L (Table 2). For lymphocyte counts up to 6 × 10^9^/L (<6 × 10^9^/L), higher values are associated with a lower risk of PRD. For lymphocyte counts equal to or greater than 6 × 10^9^/L, increasing lymphocyte counts are associated with a higher risk of PRD.

After adding treatment information to the predictor set, besides male sex, leukocyte count, and lymphocyte count, age and treatment also predicted PRD risk. Older age was associated with an increasing PRD risk. Receiving >4 cycles of ABVD (without radiotherapy) or ABVD plus radiotherapy were associated with lower PRD risk compared with ≤4 cycles of ABVD without radiotherapy, where the reduction in PRD risk when receiving ABVD plus radiotherapy was time-dependent and stronger in the first 9 months after diagnosis (Table 2, Supplemental Figure S7-1).

For advanced stage patients, higher albumin was associated with lower PRD risk, while older age (especially age > 35 years) was associated with higher PRD risk (Table 3). An increased leukocyte count showed a complex non-linear relation with PRD risk, where PRD risk increased for values from ≥10 to <20 × 10^9^/L while PRD risk decreased otherwise.

In the models including treatment information, compared with ≤6 ABVD cycles, receipt of >6 ABVD cycles was associated with lower PRD risk up to 8 months after diagnosis, while it appeared to increase PRD risk from 8 to 17 months, after which the association with PRD risk almost disappeared. Receipt of BEACOPP (regardless of the number of cycles) compared with ≤6 ABVD cycles was associated with lower risk of PRD, but the decrease in risk became smaller at 8 months and subsequently at 17 months after diagnosis (see Supplemental Figure S7-2 for an illustration of time dependent effects).

### 3.2. Validation of Models for Progression-Free Survival

Among the Danish HL patients diagnosed between 2000 and 2018, 998 (59.6%) patients had early-stage and 677 (40.4%) advanced-stage disease (Supplemental Table S8). In total, 451 patients (207 early-stage and 244 advanced-stage patients) progressed, had a relapse or died within 5 years of diagnosis, while 116 patients in early stage and 137 advanced-stage patients died during follow-up.

Validation of our early-stage models in Danish patients diagnosed between 2008 and 2018, showed IPCW AUC values for the prognostic model of 0.64 (95% CI: 0.55–0.73) at 3 years and 0.63 (95% CI: 0.55–0.72) at 5 years. The IPCW AUC increased to 0.71 (95% CI: 0.62–0.80, *p*-value = 0.08) at 3 years and 0.71 (95% CI: 0.63–0.79, *p*-value = 0.04) at 5 years in the treatment-based model.

**Figure 1:**
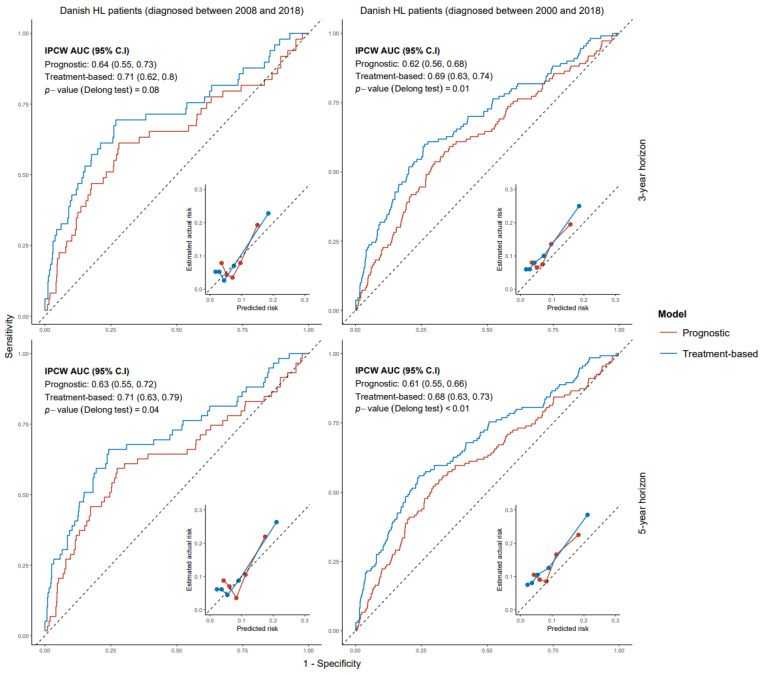
ROC-Calibration plots of early-stage models for progression-free survival on Danish HL patients. ROC: receiver operating characteristic. HL: Hodgkin lymphoma.

**Figure 2:**
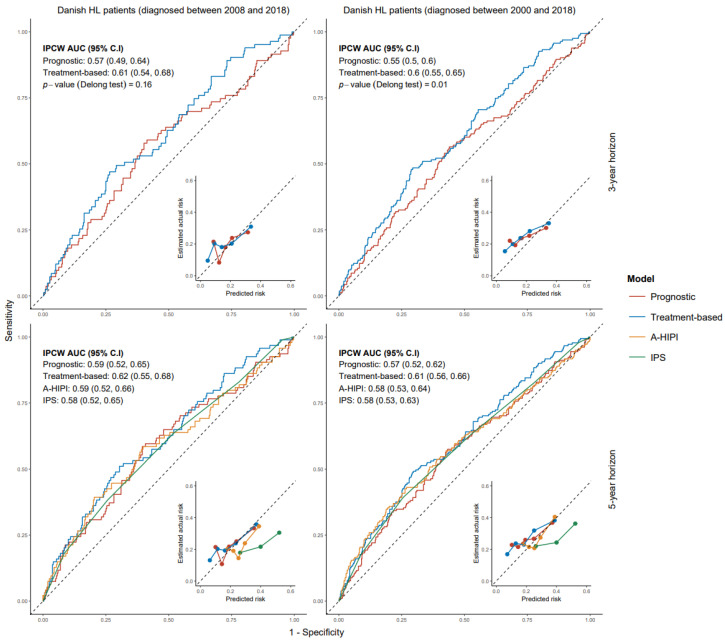
ROC-Calibration plots of advanced-stage models for progression-free survival on Danish HL patients. ROC: receiver operating characteristic. HL: Hodgkin lymphoma. A-HIPI: Advanced-Stage Hodgkin International Prognostic Index. IPS: International Prognostic Index.

In Danish patients diagnosed in 2008–2018, IPCW AUC values for advanced-stage prognostic and treatment-based models were 0.57 (95% CI: 0.49–0.64) and 0.61 (95% CI: 0.54–0.68), respectively, at the 3-year horizon (*p*-value = 0.16). At the 5-year horizon, IPCW AUC values for advanced stage prognostic and treatment-based models were 0.59 (95% CI: 0.52–0.65) and 0.62 (95% CI: 0.55–0.68), respectively (*p*-value = 0.33). The IPCW AUC for IPS and A-HIPI at the 5-year horizon were 0.58 (95% CI: 0.52–0.65) and 0.59 (95% CI: 0.52–0.66), respectively. There was no significant difference between the IPCW AUC values of IPS and A-HIPI and those of our advanced stage models. Supplemental Tables S9-1 to S9-4 provide detailed validation results and also show model comparisons on Danish patients diagnosed between 2000 and 2018. All models were reasonably well calibrated except for IPS, which overestimated PRD risk. Figure 1 and Figure 2 provide ROC-Calibration curves for early and advanced stage models, respectively.

**Figure 3:**
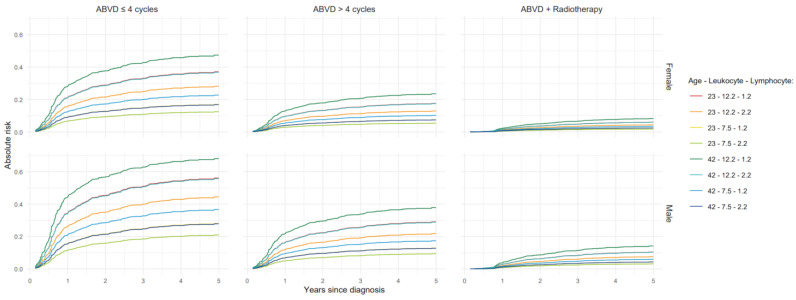
Absolute risk estimates up to 5 years after diagnosis based on a treatment-based model for early-stage HL patients. HL: Hodgkin lymphoma. The data are represented at the 1st and 3rd quartile cut-off values of age in years, leukocyte count ($\times 10^{9}$/L), and lymphocyte count ($\times 10^{9}$/L).

**Figure 4:**
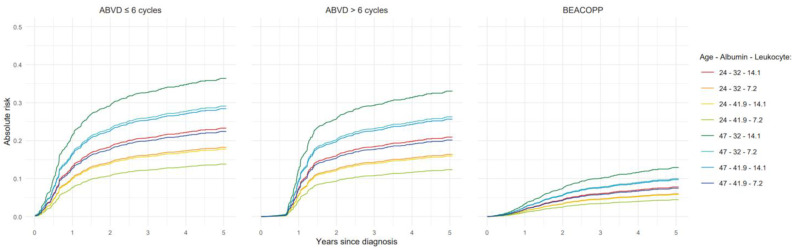
Absolute risk estimates up to 5 years after diagnosis based on treatment-based model for advanced stage HL patients. HL: Hodgkin lymphoma. The data are represented at the 1st and 3rd quartile cut-off values of age in years, albumin in gr/L, and leukocyte count ($\times 10^{9}$/L).

Furthermore, Figure 3 and Figure 4 show absolute risk estimates for the treatment-based models up to 5 years after diagnosis, with values for continuous predictors fixed at their respective 1st and 3rd quartiles.

### 3.3. Models for All-Cause Mortality

In early stages, male sex, abnormal LDH, and older age were associated with increased risk of death in which the increased risk due to age was stronger for ages ≥35 years. In a model with treatment information, receiving >4 ABVD cycles without radiotherapy or ABVD + radiotherapy showed a lower mortality risk compared with ≤4 ABVD cycles without radiotherapy, where the reduction in risk by ABVD + radiotherapy was stronger during the first 24 months after diagnosis (Supplemental Figure S7-1). In Danish patients diagnosed between 2000 and 2018, the 5-year IPCW AUC of the prognostic model was 0.58 (95% CI: 0.49–0.68) and significantly lower than the IPCW (0.68, 95% CI: 0.60–0.77) for the treatment-based model.

In advanced stages, male gender and older age predicted higher mortality risk with the effect of age being stronger for patients ≥40 years. Albumin levels and leukocyte counts showed complex non-linear and time-dependent associations with mortality risk. The IPCW AUC for both models with and without treatment effect at the 5-year prediction horizon was 0.70 (95% CI: 0.63–0.76) on Danish patients diagnosed between 2000 and 2018. Although adding treatment resulted in a significant treatment effect on mortality risk, the predictive performance of the treatment-based model remained the same (see Supplement S10).

## 4. Discussion

This is the first study to incorporate pre-treatment prognostic factors as well as contemporary treatments to predict risk of progression, relapse or death for early and advanced HL patients up to 5 years after diagnosis. This is a crucial step towards the development of models enabling the weighing of relapse risk versus treatment-specific risks of late complications of treatment. Our treatment-based models generally had better discriminatory ability than our prognostic models without treatment. Our new models validated well in an external cohort of Danish HL patients.

Our study shows that in early-stage HL, adding radiotherapy to ABVD chemotherapy is associated with a reduced risk of PRD and lower all-cause mortality. A systematic review with meta-analysis of randomized controlled trials executed between 1980 and 2009, comparing chemotherapy alone versus the same chemotherapy regimen plus radiotherapy, also showed that the addition of radiotherapy in early stages is associated with better tumor control and better overall survival of HL patients [19]. Furthermore, the recently published joint analysis of the EORTC/LYSA/FIL H10 and NCRI RAPID trials showed that radiotherapy significantly reduced risk of early relapse in patients who achieved an early metabolic complete response on chemotherapy [20]. For the advanced stage of disease, we observed that (escalated) BEACOPP was clearly superior over ABVD in terms of risk of PRD. A pooled analysis of four randomized trials from the U.S. and Europe comparing BEACOPP variants with ABVD in advanced-stage disease also showed a clear progression-free survival advantage and slightly improved overall survival of BEACOPP over ABVD up to 7 years after treatment [21]. An Italian randomized trial comparing ABVD vs. BEACOPP in 331 patients with stage IIB, III or IV newly diagnosed HL, also showed a significantly better rate of freedom from first progression and better initial tumor control in patients treated with BEACOPP [22].

The benefit of including radiotherapy in early stages and treating advanced stage patients with BEACOPP instead of ABVD should not be interpreted as evidence for the under-treatment of patients who received other treatments. Even though these treatments appear to be associated with lower risk of first relapse and lower all-cause mortality, they may be accompanied by more short-term side effects and higher risks of various late adverse events, including second malignancies and cardiovascular diseases [23]. The associations of radiation with the occurrence of second malignancies, various cardiovascular diseases and endocrinopathies have been well studied and summarized in previous publications [23]. BEACOPP-like chemotherapy is also associated with significant acute hematological and non-hematological toxicities, higher risk of infertility and possibly increased risk of second malignancies [24]. Importantly, a recent publication showed a better risk-to-benefit ratio of brentuximab vedotin, etoposide, cyclophosphamide, doxorubicin, dacarbazine, and dexamethasone (BrECADD) compared to BEACOPP escalated in patients with advanced-stage HL [25]. Combining estimated relapse risks from our models with estimated risks for other late effects and possibly patient-reported outcomes (such as quality of life scores) is necessary to weigh the risk of relapse against late complications and an HL survivor’s quality of life so a proper decision on the choice of treatment can be made.

Our data are population-based in nature and most patients were not treated in randomized clinical trials. Hence, we cannot conclude that the boost in predictive performance of models with treatments information is completely due to the described variation in treatments and it may (at least partly) reflect the pre-treatment (baseline) clinical factors that affected the choice of treatment.

A few variables that showed predictive value in other studies and that are used in clinical practice to determine the treatment policy [2] were not available in our data, including the presence of bulky disease, the erythrocyte sedimentation rate (ESR), presence of comorbidities and response to chemotherapy. For example, in early-stage patients, bulky mediastinal disease is considered as a risk factor and especially in case of the presence of B-symptoms, patients may be treated primarily with chemotherapy instead of combined modality treatment [26]. Therefore, a major part of the variation explained by our treatment variables in early stages might have been explained by the information about the presence of bulky disease. However, for advanced-stage patients, information on the presence of bulky disease or abnormal ESR would unlikely have improved our prognostic model to such an extent that these variables would explain the better performance of a model including treatment information. Bulky disease or abnormal ESR were, for instance, available in the study published by the HoLISTIC consortium [10] but the predictive performance of their recently published A-HIPI model was comparable to the performance of our advanced-stage prognostic model without treatment information. Information from response evaluation using PET scans may, however, prove to be an important prognostic and predictive factor. A report from a joint Italian-Danish study on advanced-stage patients treated with ABVD showed that PET scan results in advanced stages of disease almost overshadowed the value of the IPS [27]. Information of interim PET scans, which nowadays is used to individualize treatment [2] could further boost the predictive performance of our prognostic models. Other predictors, which could improve the prediction of relapse risk but were not available for our population, are characteristics of Hodgkin and Reed–Sternberg (HRS) cells, information regarding the tumor microenvironment, genetic background of patients and three-dimensional telomere signatures of HRS cells at diagnosis [28,29]. Nevertheless, inclusion of information on further potential predictors in the future will make it possible to evaluate possible gains in the predictive performance of the model.

Our study has some other limitations besides the lack of information on some potentially predictive variables. We did not have information on the cause of death, and we therefore only could predict all-cause mortality. Importantly, the limited number of deaths in our cohort (6.3% in total by the end of follow-up) did result in imprecise estimates of treatment effects over time (see Supplement S10), which may explain why adding treatment to the model predicting mortality in advanced-stage patients resulted in a significant treatment effect on mortality risk, while the predictive performance of the treatment-based model did not improve compared to the prognostic model. Furthermore, since many deaths within 5 years of HL diagnosis were HL-related and following a progression or relapse, combining progression/relapse with deaths within 5 years of HL diagnosis, created a dataset with more events, allowing the better detection of relationships between predictors and PRD. Access to a larger dataset with higher number of cause-specific events (i.e., progression, relapse or HL-specific mortality) can provide greater insight, especially through competing risk analysis. Additionally, although we studied relatively recently treated patients, treatment policies have already changed since then, with current patients usually receiving response-adapted therapy, and new agents such as antibody-drug conjugates (i.e., brentuximab vedotin) and immune check point inhibitors (i.e., nivolumab and pembrolizumab) are being introduced in first line treatment [1,25,30,31].

Our models were developed and validated using homogeneous HL populations, comprising patients largely of northern European descent (i.e., HL patients living in the Netherlands and Denmark) and comprised individuals aged 15–60 years at the time of HL diagnosis. Our models may not validate well in pediatric HL populations. Further validations of our models in other populations which may include patients over 60 years of age or those treated in different geographic regions of the world will be essential to assess the generalizability of these models to broader populations.

Although current risk estimators may become less relevant due to the emergence of new treatment policies, data on these regimens are mainly based on results from clinical trials and long term follow up is still limited. We have, however, developed a robust and dynamic platform that can be promptly updated and validated as more current treatment information becomes available in the near future.

## 5. Conclusions

We developed well-calibrated prognostic models (without treatment information) that can be used to estimate the absolute risk of first relapse for early- and advanced-stage HL patients with good performance. Moreover, the incorporation of stage-specific HL treatment information in our models created well-calibrated treatment confirmation tools that have at least equal and often better predictive performance over our models only including pre-treatment prognostic factors. These models may help to weigh the benefits of contemporary treatments and the associated risks of late adverse effects.

## Figures and Tables

**Table 1:** Prognostic and treatment information of Dutch HL patient population (N = 2340).

	Year of Diagnosis: 2008–2010		Year of Diagnosis: 2014–2018		Year of Diagnosis: 2008–2018	
	Early Stage (N = 503)	Advanced Stage (N = 345)	Early Stage (N = 834)	Advanced Stage (N = 658)	Early Stage (N = 1337)	Advanced Stage (N = 1003)
**Sex**						
female	261 (51.9%)	127 (36.8%)	412 (49.4%)	233 (35.4%)	673 (50.3%)	360 (35.9%)
male	242 (48.1%)	218 (63.2%)	422 (50.6%)	425 (64.6%)	664 (49.7%)	643 (64.1%)
**Age, years**						
Mean (SD)	33.4 (12.6)	36.3 (13.4)	33.1 (12.0)	34.9 (13.0)	33.2 (12.2)	35.3 (13.1)
Median [Min, Max]	31.0 [15.0, 60.0]	35.0 [15.0, 60.0]	31.0 [15.0, 60.0]	33.0 [15.0, 60.0]	31.0 [15.0, 60.0]	34.0 [15.0, 60.0]
15–18	41 (8.2%)	35 (10.1%)	73 (8.8%)	63 (9.6%)	114 (8.5%)	98 (9.8%)
19–29	199 (39.6%)	91 (26.4%)	319 (38.2%)	207 (31.5%)	518 (38.7%)	298 (29.7%)
30–39	104 (20.7%)	78 (22.6%)	190 (22.8%)	164 (24.9%)	294 (22.0%)	242 (24.1%)
40–49	77 (15.3%)	66 (19.1%)	144 (17.3%)	99 (15.0%)	221 (16.5%)	165 (16.5%)
50–60	82 (16.3%)	75 (21.7%)	108 (12.9%)	125 (19.0%)	190 (14.2%)	200 (19.9%)
**Ann arbor stage**						
I	86 (17.1%)	-	138 (16.5%)	-	224 (16.8%)	-
II	417 (82.9%)	-	696 (83.5%)	-	1113 (83.2%)	-
III	-	204 (59.1%)	-	268 (40.7%)	-	472 (47.1%)
IV	-	141 (40.9%)	-	390 (59.3%)	-	531 (52.9%)
**Presence of B-symptoms**						
no	369 (73.4%)	126 (36.5%)	599 (71.8%)	253 (38.4%)	968 (72.4%)	379 (37.8%)
yes	133 (26.4%)	215 (62.3%)	216 (25.9%)	390 (59.3%)	349 (26.1%)	605 (60.3%)
Missing	1 (0.2%)	4 (1.2%)	19 (2.3%)	15 (2.3%)	20 (1.5%)	19 (1.9%)
**Extra-nodal disease**						
no	489 (97.2%)	194 (56.2%)	792 (95.0%)	240 (36.5%)	1281 (95.8%)	434 (43.3%)
yes	14 (2.8%)	151 (43.8%)	41 (4.9%)	418 (63.5%)	55 (4.1%)	569 (56.7%)
Missing	0 (0%)	0 (0%)	1 (0.1%)	0 (0%)	1 (0.1%)	0 (0%)
**LDH**						
normal	336 (66.8%)	195 (56.5%)	661 (79.3%)	433 (65.8%)	997 (74.6%)	628 (62.6%)
below/above standard limits	98 (19.5%)	94 (27.2%)	158 (18.9%)	217 (33.0%)	256 (19.1%)	311 (31.0%)
Missing	69 (13.7%)	56 (16.2%)	15 (1.8%)	8 (1.2%)	84 (6.3%)	64 (6.4%)
**Number of involved nodes**						
0–4	408 (81.1%)	77 (22.3%)	655 (78.5%)	154 (23.4%)	1063 (79.5%)	231 (23.0%)
≥4	80 (15.9%)	248 (71.9%)	179 (21.5%)	504 (76.6%)	259 (19.4%)	752 (75.0%)
Missing	15 (3.0%)	20 (5.8%)	0 (0%)	0 (0%)	15 (1.1%)	20 (2.0%)
**Hemoglobin, mmol/L**						
Mean (SD)	8.18 (1.08)	7.48 (1.16)	8.18 (1.14)	7.49 (1.31)	8.18 (1.12)	7.48 (1.26)
Median [Min, Max]	8.20 [4.60, 11.3]	7.50 [3.90, 10.9]	8.20 [3.30, 10.9]	7.60 [1.90, 11.1]	8.20 [3.30, 11.3]	7.60 [1.90, 11.1]
Missing	53 (10.5%)	33 (9.6%)	37 (4.4%)	14 (2.1%)	90 (6.7%)	47 (4.7%)
**Albumin, gr/L**						
Mean (SD)	40.9 (5.72)	36.6 (7.01)	40.5 (5.69)	36.5 (6.69)	40.6 (5.70)	36.5 (6.79)
Median [Min, Max]	41.4 [17.0, 52.0]	38.0 [7.00, 50.0]	41.0 [4.40, 53.0]	37.0 [14.0, 52.9]	41.0 [4.40, 53.0]	37.0 [7.00, 52.9]
Missing	142 (28.2%)	92 (26.7%)	146 (17.5%)	78 (11.9%)	288 (21.5%)	170 (16.9%)
**Leukocyte,** **×10^9^** **/L**						
Mean (SD)	10.3 (4.13)	10.9 (5.67)	10.4 (4.16)	11.3 (5.68)	10.3 (4.15)	11.2 (5.68)
Median [Min, Max]	9.50 [0.600, 27.1]	10.3 [0.500, 39.3]	9.60 [0.700, 34.3]	10.7 [0.800, 36.3]	9.50 [0.600, 34.3]	10.5 [0.500, 39.3]
Missing	42 (8.4%)	31 (9.0%)	33 (4.0%)	10 (1.5%)	75 (5.6%)	41 (4.1%)
**Lymphocyte,** **×10^9^** **/L**						
Mean (SD)	2.41 (3.97)	1.71 (2.10)	2.09 (2.92)	1.69 (1.87)	2.20 (3.31)	1.70 (1.94)
Median [Min, Max]	1.60 [0.300, 37.9]	1.40 [0.100, 23.4]	1.70 [0.200, 37.4]	1.40 [0.100, 25.4]	1.60 [0.200, 37.9]	1.40 [0.100, 25.4]
Missing	135 (26.8%)	92 (26.7%)	86 (10.3%)	42 (6.4%)	221 (16.5%)	134 (13.4%)
**Primary treatment**						
ABVD ≤ 4 cycles (no RT)	53 (10.5%)	-	51 (6.1%)	-	104 (7.8%)	-
ABVD > 4 cycles (no RT)	60 (11.9%)	-	133 (15.9%)	-	193 (14.4%)	-
ABVD + RT	332 (66.0%)	-	518 (62.1%)	-	850 (63.6%)	-
ABVD ≤ 6 (w/wo RT)	-	139 (40.3%)	-	254 (38.6%)	-	393 (39.2%)
ABVD > 6 (w/wo RT)	-	128 (37.1%)	-	104 (15.8%)	-	232 (23.1%)
(Escalated) BEACOPP (w/wo RT)	-	54 (15.7%)	-	248 (37.7%)	-	302 (30.1%)
other	58 (11.5%)	24 (7.0%)	132 (15.8%)	52 (7.9%)	190 (14.2%)	76 (7.6%)

w/wo: with/without; RT: radiotherapy. Patients aged ≤18 years were often treated under childhood protocols; 78% (*n* = 63) of those ≤18 years with early-stage disease and 74% (*n* = 51) of those ≤18 years with advanced-stage disease were classified in the “Other” treatment category.

**Table 2:** Final models for progression-free survival in early stages based on prognostic factors with and without treatment.

		Prognostic Model				Treatment-Based Model			
Variable	Category/Spline Term	Estimate	Std Error	*p*-Value	PH-*p*	Estimate	Std Error	*p*-Value	PH-*p*
Age, years	-	-	-	-	-	0.017	0.007	0.017	0.552
Sex	female	Reference				Reference			
	male	0.460	0.178	0.009	0.188	0.579	0.181	0.001	0.294
Leukocyte, ×$10^{9}$/L	max(0, 12−leukocyte)	−0.204	0.049	<0.001	0.612	−0.207	0.049	<0.001	0.592
	max(0, leukocyte−12)	−0.015	0.037		0.243	0.002	0.036		0.310
Lymphocyte, ×$10^{9}$/L	max(0, 1−lymphocyte)	0.914	0.701	<0.001	0.062	1.061	0.733	0.023	0.107
	max(0, lymphocyte−1)	−0.371	0.163		0.116	−0.345	0.172		0.228
	max(0, lymphocyte−6)	0.422	0.200		0.274	0.407	0.210		0.376
Primary Treatment	ABVD ≤ 4 cycles (no RT)	Reference				Reference			
	ABVD > 4 cycles (no RT)	-	-	-	-	−0.869	0.261	<0.001	0.297
	ABVD+RT _≤ 9 months_	-	-		-	−3.822	0.620		0.983
	ABVD+RT _> 9 months_	-	-		-	−1.560	0.264		0.075
	other	-	-		-	−1.216	0.286		0.349
		Global PH *p*-value: 0.132				Global PH *p*-value: 0.061			

RT: radiotherapy; PH: proportional hazards; PH-*p*: *p*-value of PH assumption test; Std: standard. The reported *p*-values are for the terms before the possible creation of time-dependent coefficients. Time (in months) in subscript following a term indicates the effect of the term in that specific time frame. max(0,x−a) creates the linear effect of x for values greater than a (a knot). max(0,a−x) creates the linear effect for values smaller than a. Since this creates a decreasing term with respect to any increase in x, a negative coefficient estimate translates to an increased risk while a positive coefficient translates to a decreased risk.

**Table 3:** Final models for progression-free survival in advanced stages based on prognostic factors with and without treatment.

		Prognostic Model				Treatment-Based Model			
Variable	Category/Spline Term	Estimate	Std Error	*p*-Value	PH-*p*	Estimate	Std Error	*p*-Value	PH-*p*
Age, years	max(0, 35−Age)	−0.020	0.015	<0.001	0.641	−0.029	0.016	<0.001	0.882
	max(0,Age−35)	0.027	0.010		0.729	0.018	0.010		0.866
Albumin, gr/L	-	−0.027	0.011	0.019	0.078	−0.030	0.011	0.005	0.117
Leukocyte, ×$10^{9}$/L	max(0, 10−Leukocyte)	0.095	0.039	<0.001	0.391	0.077	0.039	<0.001	0.387
	max(0, Leukocyte−10)	0.127	0.029		0.714	0.120	0.029		0.801
	max(0, Leukocyte−20)	−0.216	0.068		0.831	−0.187	0.067		0.900
Primary Treatment	ABVD ≤ 6 cycles	Reference				Reference			
	ABVD > 6 cycles _≤ 8 months_	-	-	-	-	−3.341	1.010	<0.001	0.148
	ABVD > 6 cycles _8–17 months_	-	-		-	0.690	0.247		0.685
	ABVD > 6 cycles _> 17 months_	-	-		-	−0.029	0.296		0.841
	(Escalated) BEACOPP _≤ 8 months_	-	-		-	−2.445	0.596		0.972
	(Escalated) BEACOPP _8–17 months_	-	-		-	−1.220	0.420		0.772
	(Escalated) BEACOPP _> 17 months_	-	-		-	−0.546	0.315		0.854
	other	-	-		-	0.045	0.291		0.939
		Global PH *p*-value: 0.470				Global PH *p*-value: 0.932			

All primary treatment categories in advanced stages are represented irrespective of radiotherapy (with/without radiotherapy); PH: proportional hazards; PH-*p*: *p*-value of PH assumption test; Std: standard. Reported *p*-values are for the terms before the possible creation of time-dependent coefficients. Time (in months) in subscript following a term indicates the effect of the term in that specific time frame. max(0,x−a) creates the linear effect of x for values greater than a (a knot). max(0,a−x) creates the linear effect for values smaller than a. Since this creates a decreasing term with respect to any increase in x, a negative coefficient estimate translates to an increased risk while a positive coefficient translates to a decreased risk.

## Data Availability

Restrictions apply to the availability of these data. Data were obtained from The Netherlands Cancer Registry (NCR) and Copenhagen University Hospital and are available from the corresponding authors with the permission of the NCR and Copenhagen University Hospital.

## Supplementary Materials

The following supporting information can be downloaded at: https://www.mdpi.com/article/10.3390/cancers17172760/s1, Table S1-1: Regimens/drugs combinations or terms that were considered as “other” in early stages; Table S1-2: Regimens/drugs combinations or terms that were considered as “other” in advanced stages; Figure S3: Association strength values among available predictors; Figure S4: Functional form of predictors for different outcomes in early and advanced stage patients; Figure S7-1: Time-dependent effects for progression-free and overall survival in early stages; Figure S7-2: Time-dependent effects for progression-free and overall survival in advanced stages; Table S8: Prognostic and treatment information of Danish HL patients (used for validations); Table S9-1: Validation results of early stage models for progression-free survival; Table S9-2: Comparison of early stage models for progression-free survival by Delong test; Table S9-3: Validation results of advanced stage models for progression-free survival; Table S9-4: Comparison of advanced stage models for progression-free survival by Delong test; Table S10-1: Final prognostic and treatment-based models for overall survival in early stages; Table S10-2: Final prognostic and treatment-based models for overall survival in advanced stages; Figure S10-1: ROC-Calibration curves of overall survival models in early stages; Table S10-3: Validation results of early stages models for overall survival; Table S10-4: Comparison of early stage models for overall survival based on Delong test; Figure S10-2: ROC-Calibration curves of overall survival models in advanced stages; Table S10-5: Validation results of advanced stages models for overall survival; Table S10-6: Comparison of advanced stage models for overall survival based on Delong test; Figure S10-3: Effect of treatment on death from all causes through time on Dutch and Danish HL patients.

## Abbreviations

The following abbreviations are used in this manuscript:

HL	Hodgkin Lymphoma
GHSG	German Hodgkin Study Group
EORTC	European Organization for Research and Treatment of Cancer
IPS	International Prognostic Score
A-HIPI	Advanced-Stage Hodgkin Lymphoma International Prognostic Index
ABVD	Adriamycin (Doxorubicin), Bleomycin, Vinblastine, and Dacarbazine
BEACOPP	Bleomycin, Etoposide, Doxorubicin, Cyclophosphamide, Vincristine, Procarbazine, Prednisone
PRD	Progression, Relapse or Death
PFS	Progression-Free Survival
OS	Overall Survival
PH	Proportional Hazards
LOESS	Locally Estimated Scatterplot Smoothing
IPCW	Inverse Probability of Censoring Weighting
AUC	Area Under the (ROC) Curve
IQR	Inter Quantile Range
CI	Confidence Interval
BrECADD	Brentuximab vedotin, Etoposide, Cyclophosphamide, Doxorubicin, Dacarbazine and Dexamethasone
ESR	Erythrocyte Sedimentation Rate
