# Supplementary material for: Predicting Absolute Risk of First Relapse in Classical Hodgkin Lymphoma by Incorporating Contemporary Treatment Effects
Source: Cancers (Basel). 2025 Aug 24;17(17):2760. doi: 10.3390/cancers17172760 (PMC12427322; doi:10.3390/cancers17172760)
Supplement: Supplementary file 1 [file cancers-17-02760-s001.zip › cancers-3787236-supplementary.pdf]

Supplements:

S1: Stage-specific treatment variables

Primary chemotherapy treatment plans consisted of different combinations of regimens. The most frequently applied chemotherapy regimens were ABVD & (Escalated) BEACOPP. A variety of other regimens was used and these regimens were all combined in the "other treatments" category.

The treatment categories for early stages of disease were created as follows:

1. If a patient had received ABVD (regardless of other involved regimens/drugs in the plan except for BEACOPP), the treatment for that patient was labeled as “ABVD” otherwise, it was labeled as “other”. This means that for early stages, for patients, who were treated with (Escalated) BEACOPP, treatment was categorized as “other”.

Table S1-1: Regimens/drugs combinations or terms that were considered as “other” in early stages

Combinations	Frequency
ABVD + BEACOPP + External radiotherapy	49
BEACOPP	42
COPDAC + OEPA	32
ABVD + BEACOPP	12
OEPA	7
COPDAC + OEPA + External radiotherapy	7
Systemic chemotherapy	6
External radiotherapy	5
BEACOPP + External radiotherapy	3
AVD + External radiotherapy	2
Systemic chemotherapy + External radiotherapy	2
ABVD + BEACOPP + rituximab + External radiotherapy; Adriamycin + Bleomycin + CEOP + External radiotherapy; Adriamycin + Bleomycin + CVP + etoposide; Anthracycline containing chemotherapy + External radiotherapy; AVD; BEACOPP + rituximab; BEACOPP + rituximab + External radiotherapy; Bleomycin + Cyclophosphamide + OEPA + External radiotherapy; ChlVPP + External radiotherapy; CHOEP + CHOP21 + rituximab; CHOP14 + External radiotherapy; CHOP21 + rituximab; COPAD; COPADM + OEPA; EBVP; Intensive chemotherapy; LOPP + External radiotherapy; OEPA + External radiotherapy; OPA + Procarbazine + External radiotherapy; PECC; PECC + External radiotherapy; rituximab; External radiotherapy + VIDE	Each once

2. We divided ABVD into two groups, based on whether a patient also received radiotherapy, into "ABVD" or "ABVD+RT". When a patient was categorized into "ABVD+RT" the number of ABVD cycles was not take into account.
3. The "ABVD" category was further subdivided into ≤ 4 cycles & >4 cycle of ABVD based on the median number of ABVD cycles in early stages.

These steps resulted in 4 categories labeled "ABVD≤ 4 cycles", "ABVD>4 cycles", "ABVD+RT" & "other" for early stage patients.

For advanced stage patients, treatment was categorized as follows:

1. If a patient had received BEACOPP or Escalated BEACOPP the treatment category was set to "(Escalated) BEACOPP", irrespective of whether a patient had also received one or more cycles of ABVD. This was done because (Escalated) BEACOPP is considered to be a more intense regimen compared to ABVD and in cases where both ABVD & (Escalated) BEACOPP were given together, the number of ABVD cycles was usually low.
2. For the remaining patients, all patients who only received ABVD treatment was categorized as "ABVD", otherwise treatment was categorized as "other" treatment.

Table S1-2: Regimens/drugs combinations or terms that were considered as "other" in advanced stages

**Combination**	**Frequency**
COPDAC + OEPA	22
COPDAC + OEPA + External radiotherapy	6
Systemic chemotherapy	6
OEPA	5
Systemic chemotherapy + External radiotherapy	5
CHOP21	4
Intensive chemotherapy	3
Brentuximab vedotin + CDE + Dacarbazine; EBVP + MOPP; MOPP/ABV; OEPA + External radiotherapy; Rituximab	Each twice
Autologous SCT + DHAP + VIM; AVD; AVD + MOPP; AVD + Rituximab; BrECADD; Brentuximab vedotin + CDE; CEOP; ChlVPP; CHOP14; COPDAC + OEPA + Systemic chemotherapy; COPP + OEPA + External radiotherapy; Dexamethasone + Etoposide + Rituximab; DHAP; LOPP; LOPP + External radiotherapy	Each once

3. The “ABVD” category was further subdivided into ≤6 cycles & >6 cycle of ABVD based on the median number of ABVD cycles in advanced stage patients.

These steps resulted in 4 treatment categories labeled “ABVD≤ 6 cycles”, “ABVD>6 cycles”, “(Escalated) BEACOPP” & “other” for advanced stage patients.

Note: Regimens containing OEPA and/ or COPDAC were typically given to patients ≤18 years as part of childhood Hodgkin lymphoma protocols; treatment of 63 (77.8%) patients ≤18 years with early stage and 51 (74%) patients ≤18 years with advanced stage were classified in the “Other” treatment category.

S2: Steps of modeling procedure

1. Perform multiple imputation and create m imputed sets.
2. Assess functional forms of continuous predictors through LOESS curves over their values against martingale residuals of null Cox proportional hazards (PH) model and find the best form of them to be included in the model.
   - In case non-linear form was suggested in variable x, we created a piecewise linear function $h(x; c_1, c_2, \dots, c_K)$ where $c_1, c_2, \dots, c_K$ are ordered knot locations and it takes the forms $max(0, -(x - c_1))$ and $max(0, x - c_k)$ for $k = 1, \dots, K$. Number and location of knots were picked in a way that the final form best matches the LOESS curves.
3. Fit m Cox PH models using created functional forms of continuous predictors and other categorical variables on m imputed sets.
4. Using models on m imputed sets, perform p-value based variable selection where p-values are based on D2 statistic[1] of comparisons between nested models:
   a) On each imputed set, obtain deviance-based chi-square values for each predictor from comparing a nested model without that predictor with the full model. Note that predictors that are introduced by multiple terms (i.e. spline forms or dummy terms of categorical variables) are considered together.
   b) Pool chi-square values for each predictor over m imputed sets which results in D2 statistic with asymptotic F distribution for each term and then calculate p-values.
   c) Remove the predictor with the highest p-value and repeat all the steps until all p-values are less than 0.05.
   d) Pool the estimates of the remaining predictors based on Rubin's method[1] to shape the final model.
5. Test the PH assumption for final terms and also globally over imputed sets. This is done by pooling the chi-square statistic of independence tests between Schoenfeld residuals and time over imputed sets which results in D2 statistic with approximate F distribution.
6. If any PH assumptions are violated at 5% level on the final set of significant predictors, search for a set of cut-points in time to create step functions for the coefficients related to variables with non-PH effect.
   - Finding cut-points in time is done by evaluating a smooth LOESS curve over Schoenfeld residuals associated with the PH-violating terms against time.

S3: Association strength description and its values among predictors

Pairwise association strengths were measured based on predictors types. For two continuous predictors, Spearman correlation was used while Cramer's V statistic was used for two categorical variable. For continuous vs categorical variables, a regression model was fitted where the continuous and the categorical predictor were used as the dependent and independent variables, respectively and the resulting R squared was used as a measurement of the association strength.

Figure S3: Association strength values among available predictors

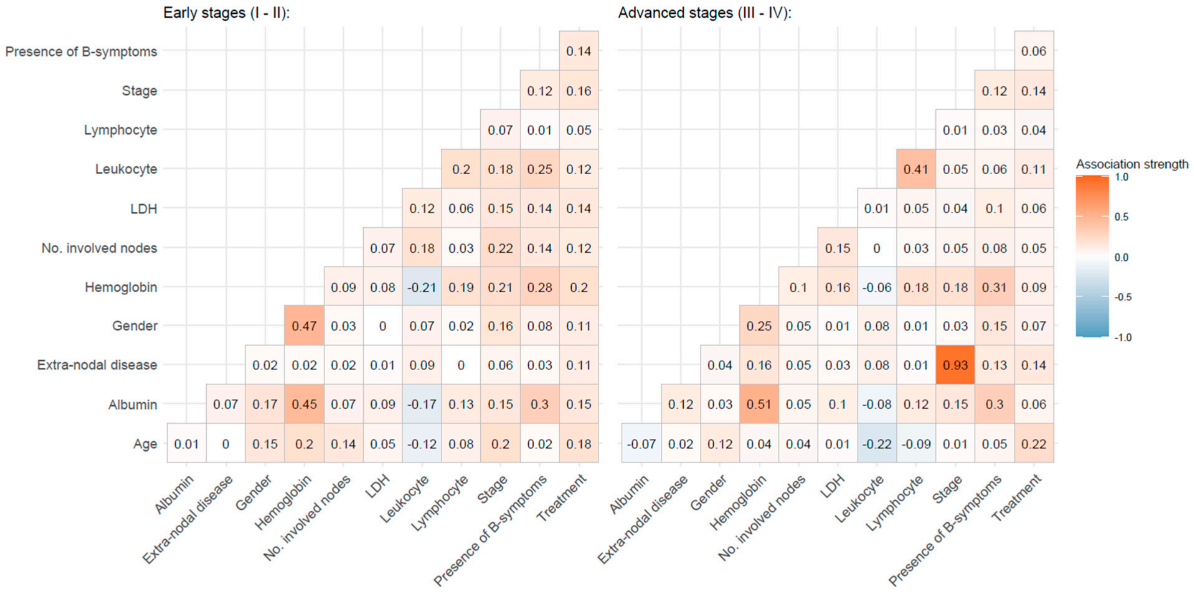

S4: Functional form of continuous predictors and the choices of non-linear forms

Figure S4: Functional form of predictors for different outcomes in early and advanced stage patients

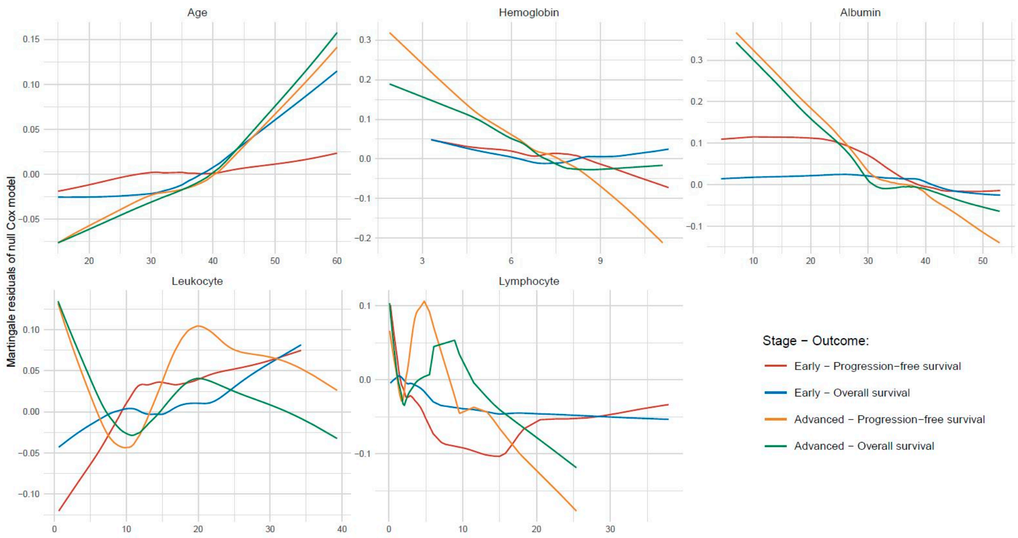

Based on the given plots, our choices for number and location of knots in linear spline forms of continuous predictors are given below.

For progression-free survival:

- **In early stage models (prognostic and treatment-based):**

  Age: linear form

  Hemoglobin: 1 knot at 8 mmol/L

  Albumin: 1 knot at 30 gr/L

  Leukocyte: 1 knot at 12 $\times 10^9/L$

  Lymphocyte: 2 knots at 1 and 6 $\times 10^9/L$

- **In advanced stage models (prognostic and treatment-based):**

  Age: 1 knot at 35 years

  Hemoglobin: Not used

  Albumin: linear form

  Leukocyte: 2 knots at 10 and 20 $\times 10^9/L$

  Lymphocyte: 2 knots at 1 and 5 $\times 10^9/L$

For overall survival:

- **In early stage models (prognostic and treatment-based):**

Age: 1 knot at 35 years

Hemoglobin: 1 knot at 7 mmol/L

Albumin: 1 knot at 40 gr/L

Leukocyte: 2 knot at 12 and 22 $\times 10^9/L$

Lymphocyte: 2 knots at 1 and 6 $\times 10^9/L$

- **In advanced stage models (prognostic and treatment-based):**

  Age: 1 knot at 40 years

  Hemoglobin: not included

  Albumin: 1 knot at 32 gr/L

  Leukocyte: 2 knot at 10 and 20 $\times 10^9/L$

  Lymphocyte: 2 knots at 1 and 6 $\times 10^9/L$

S5: Details of absolute risk estimation

We created interpolating spline functions (*splinefun* function in R) between time and the estimated cumulative hazard estimates in each imputed dataset to shape the cumulative hazard function for each individual. Estimated risks for each individual from each imputed data were then averaged (Rubin's method[1]) to generate the final individual risk. Even though this approach is practical, it's not repeatable in the future for those without access to our model objects in R. In order to provide our risk estimators in a way that is usable by anyone in any platform, we need to break down the risk estimator from a Cox model with time dependent coefficients to the cumulative baseline hazard level.

The general formula to estimate the absolute risk for an individual with predictor set $\boldsymbol{z}$ at time t from a Cox model that allows time-dependent coefficients is:

$$\hat{r}(t;\boldsymbol{z}) = 1 - \exp\left(-\widehat{\Lambda}(t;\boldsymbol{z})\right);$$

$$\widehat{\Lambda}(t;\boldsymbol{z}) = e^{\sum_{j\in P_1} z_j\beta_j} \sum_{i=1}^{S_t} \widehat{\Lambda}_0^{(i)}(\min\,(t, t_{max}^{(i)}))\, e^{\sum_{j\in P_2} z_j\beta_j^{(i)}}$$

In the formulas, S_t is the index of the time strata (among ordered time stratas) that contains t and $\widehat{\Lambda}_0^{(i)}$ and $t_{max}^{(i)}$ are the estimated cumulative baseline hazard function and the maximum time in the ith strata, respectively. Also, P_1 and P_2 are sets of indices for predictors with time independent and time dependent coefficients, respectively and $\beta_j^{(i)}$ is the effect of jth time dependent variable in the ith time strata. When there are time-dependent coefficients with the form of step functions of time, cumulative hazard estimates for an individual is the sum of cumulative hazard estimates in each stratum (made by the step function of time) at each time t.[2]

Using the given formulas and the provided pooled coefficient estimates of the different models, the only remaining part to generate absolute risks are the estimates of the cumulative baseline hazard functions. For each model, we provide these functions through regression equations with 3rd degree polynomial form of time (in years) as the predictor and the pooled cumulative baseline hazard estimates at those times as the response.

Early stage progression-free survival

Prognostic model

$0 < t < 1825\ days\ (5\ years)$:

$$\widehat{\Lambda}_0(t) \approx 0.0795 \times t - 0.0114 \times t^2 + 0.0005 \times t^3$$

Treatment-based

$0 < t \leq 270\ days\ (0.75\ year)$:

$$\widehat{\Lambda}_0^{(1)}(t) \approx -0.0015 \times t + 0.2729 \times t^2 - 0.0248 \times t^3$$

$270\ days\ (0.75\ year) < t < 1825\ days\ (5\ years)$:

$$\widehat{\Lambda}_0^{(2)}(t) \approx -0.089 + 0.157 \times t - 0.030 \times t^2 + 0.002 \times t^3$$

Advanced stage progression-free survival

Prognostic model

$0 < t < 1825\ days\ (5\ years)$:

$$\widehat{\Lambda}_0(t) \approx 0.2007 \times t - 0.0365 \times t^2 + 0.0023 \times t^3$$

Treatment-based

$0 < t \leq 240\ days\ (0.67\ year)$:

$$\widehat{\Lambda}_0^{(1)}(t) \approx 0.1406 \times t + 0.1396 \times t^2 + 0.2383 \times t^3$$

$240\ days\ (0.67\ year) < t \leq 510\ days\ (1.42\ year)$:

$$\widehat{\Lambda}_0^{(2)}(t) \approx 0.002 - 0.320 \times t + 0.619 \times t^2 - 0.222 \times t^3$$

$510\ days\ (1.42\ year) < t < 1825\ days\ (5\ years)$:

$$\widehat{\Lambda}_0^{(3)}(t) \approx -0.216 + 0.207 \times t - 0.040 \times t^2 + 0.003 \times t^3$$

Early stage all-cause mortality

Prognostic model

$0 < t < 1825\ days\ (5\ years)$:

$$\widehat{\Lambda}_0(t) \approx 0.0055 \times t - 0.0013 \times t^2 + 0.0001 \times t^3$$

Advanced stage all-cause mortality

Prognostic model

$0 < t \leq 540\ days\ (1.50\ year)$:

$$\widehat{\Lambda}_0^{(1)}(t) \approx 0.0075 \times t - 0.0038 \times t^2 + 0.0010 \times t^3$$

$540\ days\ (1.50\ year) < t < 1825\ days\ (5\ years)$:

$$\widehat{\Lambda}_0^{(2)}(t) \approx -0.00216 + 0.00118 \times t + 0.00041 \times t^2 - 0.00003 \times t^3$$

Notes:

- While our treatment-based models for overall survival are informative for investigating associations, they are not reflecting accurate treatment effects for effective prediction which is due to small number of deaths (within 5 years of Hodgkin lymphoma diagnosis) in different treatment categories (supplements S9). To prevent these models to be directly used for prediction because of the mentioned issue, we didn't provide the estimates of their cumulative baseline hazard function.
- Regression equations for a time strata that include $t = 0$ were fitted without the intercept to force them to go through the origin: $(t, cumulative\ baseline\ hazard) = (0, 0)$.

S6: Used R packages

- **openxlsx**
  Schauberger P, Walker A (2022). *openxlsx: Read, Write and Edit xlsx Files*. https://ycphs.github.io/openxlsx/index.html, https://github.com/ycphs/openxlsx.
- **tidyverse (dplyr, tidyr, purr, stringr, tibble, ggplot2, forcats)**
  Wickham H, Averick M, Bryan J, Chang W, McGowan LD, François R, Grolemund G, Hayes A, Henry L, Hester J, Kuhn M, Pedersen TL, Miller E, Bache SM, Müller K, Ooms J, Robinson D, Seidel DP, Spinu V, Takahashi K, Vaughan D, Wilke C, Woo K, Yutani H (2019). "Welcome to the tidyverse." *Journal of Open Source Software*, 4(43), 1686. doi:10.21105/joss.01686.
- **magrittr**
  Bache S, Wickham H (2022). *magrittr: A Forward-Pipe Operator for R*. https://magrittr.tidyverse.org, https://github.com/tidyverse/magrittr.
- **survival**
  Therneau T (2023). *A Package for Survival Analysis in R*. R package version 3.5-7, https://CRAN.R-project.org/package=survival.
- **riskRegression**
  Gerds T, Ohlendorff J, Ozenne B (2023). *riskRegression: Risk Regression Models and Prediction Scores for Survival Analysis with Competing Risks*. R package version 2023.12.21, https://CRAN.R-project.org/package=riskRegression.
- **caret**
  Kuhn, Max (2008). "Building Predictive Models in R Using the caret Package." *Journal of Statistical Software*, 28(5), 1–26. doi:10.18637/jss.v028.i05, https://www.jstatsoft.org/index.php/jss/article/view/v028i05.
- **recipes**
  Kuhn M, Wickham H, Hvitfeldt E (2023). *recipes: Preprocessing and Feature Engineering Steps for Modeling*. R package version 1.0.9, https://recipes.tidymodels.org/, https://github.com/tidymodels/recipes.
- **formula.tools**
  CRAN - Package formula.tools (r-project.org)
- **mice**
  van Buuren S, Groothuis-Oudshoorn K (2011). "mice: Multivariate Imputation by Chained Equations in R." Journal of Statistical Software, 45(3), 1-67. doi:10.18637/jss.v045.i03.
- **miceadds**
  Robitzsch A, Grund S (2023). miceadds: Some Additional Multiple Imputation Functions, Especially for 'mice'. R package version 3.16-18, https://CRAN.R-project.org/package=miceadds.
- **table1:**
  Rich, B. (2023). Tables of Descriptive Statistics in HTML [R package table1 version 1.4.3]. R-Project.org. https://cran.r-project.org/package=table1
- **corrplot:**
  Wei T, Simko V (2024). *R package 'corrplot': Visualization of a Correlation Matrix*. (Version 0.94), https://github.com/taiyun/corrplot.

- **ggsci:**
  Xiao N (2024). *ggsci: Scientific Journal and Sci-Fi Themed Color Palettes for 'ggplot2'*. R package version 3.2.0, https://github.com/nanxstats/ggsci, https://nanx.me/ggsci/.
- **patchwork:**
  Pedersen T (2024). *patchwork: The Composer of Plots*. R package version 1.2.0.9000, https://github.com/thomasp85/patchwork, https://patchwork.data-imaginist.com.
- **Rcompanion**
  Mangiafico SS (2024). *rcompanion: Functions to Support Extension Education Program Evaluation*. Rutgers Cooperative Extension, New Brunswick, New Jersey. version 2.4.36, https://CRAN.R-project.org/package=rcompanion/.

S7: Illustration of detected time dependent effects in the models

Figure S7-1: Time-dependent effects for progression-free and overall survival in early stages

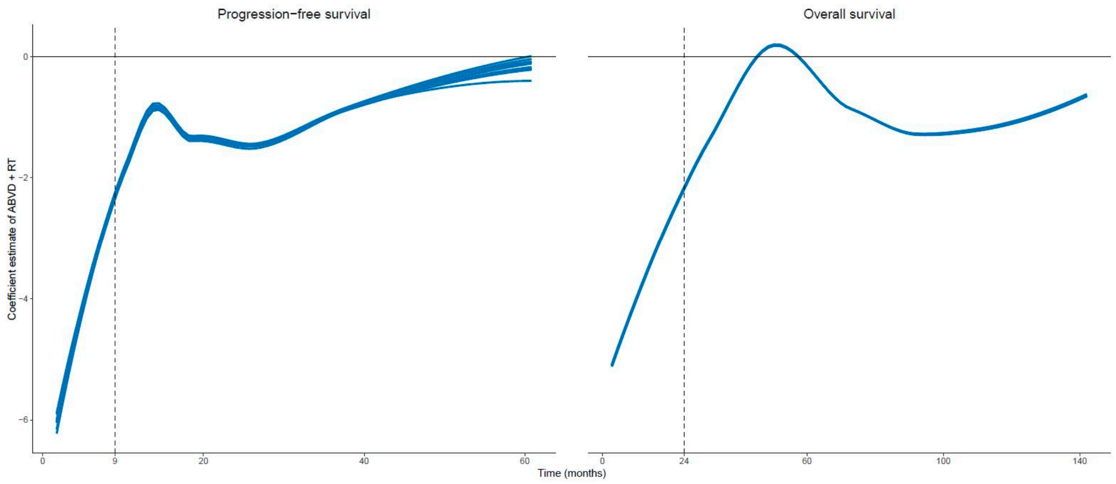

Figure S7-2: Time-dependent effects for progression-free and overall survival in advanced stages

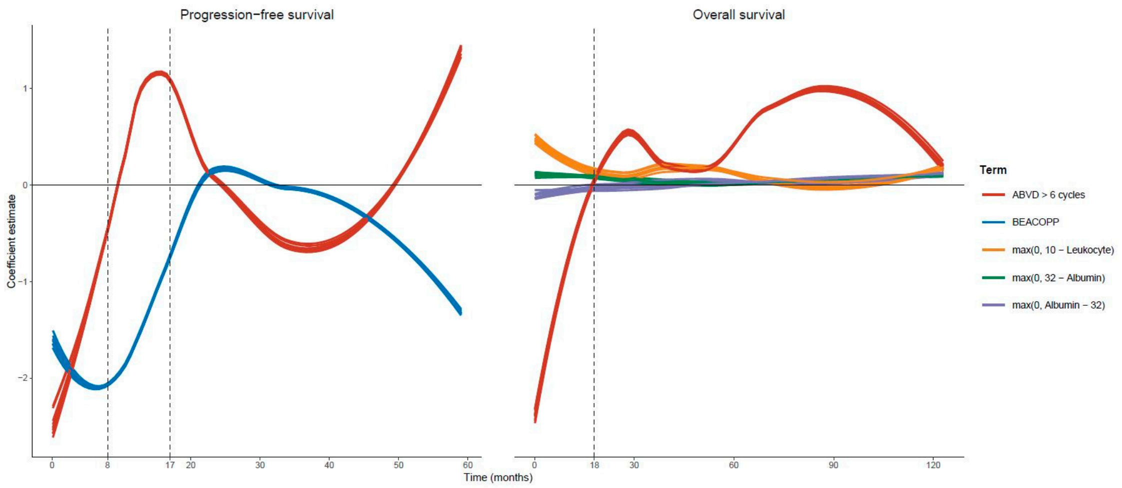

Note: Multiple lines per term refer to multiple estimates for that term from multiple imputed datasets.

S8: Descriptive table of the validation data (Danish Hodgkin lymphoma patients)

Table S8: Prognostic and treatment information of Danish HL patients (used for validations)

	Year of diagnosis: 2008-2018		Year of diagnosis: 2000-2018	
	**Early stage (N=569)**	**Advanced stage (N=419)**	**Early stage (N=998)**	**Advanced stage (N=677)**
**Sex**				
female	262 (46.0%)	178 (42.5%)	466 (46.7%)	272 (40.2%)
male	307 (54.0%)	241 (57.5%)	532 (53.3%)	405 (59.8%)
**Age (years)**				
Mean (SD)	32.8 (12.2)	36.3 (13.7)	33.7 (12.4)	36.9 (13.5)
Median [Min, Max]	30.0 [15.0, 60.0]	35.0 [15.0, 60.0]	32.0 [15.0, 60.0]	36.0 [15.0, 60.0]
**Ann arbor stage**				
I	121 (21.3%)	-	252 (25.3%)	-
II	448 (78.7%)	-	746 (74.7%)	-
III	-	199 (47.5%)	-	343 (50.7%)
IV	-	220 (52.5%)	-	334 (49.3%)
**LDH**				
normal	399 (70.1%)	199 (47.5%)	742 (74.3%)	362 (53.5%)
below/above standard limits	160 (28.1%)	215 (51.3%)	237 (23.7%)	305 (45.1%)
Missing	10 (1.8%)	5 (1.2%)	19 (1.9%)	10 (1.5%)
**Hemoglobin (mmol/L)**				
Mean (SD)	8.38 (1.07)	7.46 (1.23)	8.30 (1.13)	7.44 (1.21)
Median [Min, Max]	8.50 [4.00, 10.7]	7.50 [3.90, 10.6]	8.40 [3.20, 10.8]	7.45 [3.90, 10.6]
Missing	4 (0.7%)	0 (0%)	9 (0.9%)	1 (0.1%)
**Albumin (gr/L)**				
Mean (SD)	40.3 (5.74)	35.9 (6.81)	40.3 (5.96)	36.0 (6.70)
Median [Min, Max]	41.0 [1.29, 70.0]	37.0 [10.0, 70.0]	41.0 [1.29, 70.0]	37.0 [10.0, 70.0]
Missing	59 (10.4%)	41 (9.8%)	237 (23.7%)	117 (17.3%)
**Leukocyte (x10^9/L)**				
Mean (SD)	9.64 (3.62)	11.2 (10.2)	9.70 (4.48)	11.0 (8.76)
Median [Min, Max]	8.92 [0.500, 24.7]	9.60 [0.300, 182]	8.90 [0.500, 74.0]	9.56 [0.300, 182]

Table S8: Prognostic and treatment information of Danish HL patients (used for validations)

	Year of diagnosis: 2008-2018		Year of diagnosis: 2000-2018	
	**Early stage (N=569)**	**Advanced stage (N=419)**	**Early stage (N=998)**	**Advanced stage (N=677)**
Missing	7 (1.2%)	0 (0%)	12 (1.2%)	1 (0.1%)
**Lymphocyte (x10^9/L)**				
Mean (SD)	1.76 (0.853)	1.51 (1.22)	1.76 (1.10)	1.52 (1.31)
Median [Min, Max]	1.66 [0, 12.0]	1.36 [0, 20.0]	1.60 [0, 21.4]	1.39 [0, 20.0]
Missing	14 (2.5%)	3 (0.7%)	23 (2.3%)	8 (1.2%)
**Treatment**				
ABVD≤4 cycles (no RT)	23 (4.0%)	-	36 (3.6%)	-
ABVD>4 (no RT)	82 (14.4%)	-	125 (12.5%)	-
ABVD+RT	419 (73.6%)	-	758 (76.0%)	-
ABVD≤6 (w/wo RT)	-	177 (42.2%)	-	255 (37.7%)
ABVD>6 (w/wo RT)	-	77 (18.4%)	-	200 (29.5%)
(Escalated) BEACOPP (w/wo RT)	-	153 (36.5%)	-	194 (28.7%)
other	45 (7.9%)	12 (2.9%)	79 (7.9%)	28 (4.1%)

- w/wo: with/without; RT: radiotherapy.

S9: Validations and model comparisons for progression-free survival models on Danish HL patients

Table S9-1: Validation results of early stage models for progression-free survival

Diagnosis year	Model	Horizon	IPCW AUC	95% CI
2000-2018	Prognostic	3 years	0.621	0.562, 0.679
		5 years	0.609	0.554, 0.663
	Treatment-based	3 years	0.685	0.629, 0.742
		5 years	0.678	0.626, 0.730
2008-2018	Prognostic	3 years	0.641	0.548, 0.734
		5 years	0.635	0.549, 0.721
	Treatment-based	3 years	0.713	0.625, 0.800
		5 years	0.709	0.630, 0.788

Table S9-2: Comparison of early stage models for progression-free survival by Delong test

Diagnosis year	Horizon	Model	Reference	Delta AUC	95% CI	p-value
2000-2018	3 years	Treatment-based	Prognostic	0.065	0.014, 0.115	0.012
	5 years	Treatment-based	Prognostic	0.069	0.026, 0.113	0.002
2008-2018	3 years	Treatment-based	Prognostic	0.072	-0.010, 0.153	0.084
	5 years	Treatment-based	Prognostic	0.074	0.004, 0.144	0.039

Table S9-3: Validation results of advanced stage models for progression-free survival

Diagnosis year	Model	Horizon	IPCW AUC	95% CI
2000-2018	Prognostic	3 years	0.548	0.496, 0.600
		5 years	0.569	0.519, 0.619
	Treatment-based	3 years	0.603	0.554, 0.652
		5 years	0.608	0.560, 0.656
	A-HIPI	5 years	0.584	0.533, 0.636
	IPS	5 years	0.582	0.534, 0.631
2008-2018	Prognostic	3 years	0.566	0.494, 0.638
		5 years	0.587	0.519, 0.655
	Treatment-based	3 years	0.612	0.545, 0.678
		5 years	0.616	0.551, 0.681
	A-HIPI	5 years	0.587	0.518, 0.656
	IPS	5 years	0.585	0.519, 0.650

Table S9-4: Comparison of advanced stage models for progression-free survival by Delong test

Diagnosis year	Horizon	Model	Reference	Delta AUC	95% CI	p-value
2000-2018	3 years	Treatment-based	Prognostic	0.055	0.013, 0.097	0.011
	5 years	Treatment-based	Prognostic	0.039	0.000, 0.078	0.052
		A-HIPI	Prognostic	0.015	-0.030, 0.061	0.508
		IPS	Prognostic	0.013	-0.027, 0.053	0.519
		A-HIPI	Treatment-based	-0.024	-0.081, 0.033	0.417
		IPS	Treatment-based	-0.026	-0.082, 0.031	0.369
		IPS	A-HIPI	-0.002	-0.038, 0.034	0.901
2008-2018	3 years	Treatment-based	Prognostic	0.046	-0.018, 0.110	0.161
	5 years	Treatment-based	Prognostic	0.029	-0.030, 0.088	0.335
		A-HIPI	Prognostic	0.000	-0.062, 0.062	1.000
		IPS	Prognostic	-0.002	-0.061, 0.056	0.935
		A-HIPI	Treatment-based	-0.029	-0.112, 0.054	0.494
		IPS	Treatment-based	-0.031	-0.115, 0.053	0.463
		IPS	A-HIPI	-0.002	-0.051, 0.046	0.923

Note: IPS and A-HIPI for advanced stage patients provide risk estimates at 5 years horizon so validation and comparison at 3 years horizon is not possible.

S10: Information on overall survival models

Tables S10-1 and S10-2 are final models for overall survival in early and advanced stage HL patients.

Table S10-1: Final prognostic and treatment-based models for overall survival in early stages

		Prognostic model				Treatment-based model			
**Variable**	**Category/Spline term**	**Estimate**	**Std error**	**p-value**	**PH-p**	**Estimate**	**Std error**	**p-value**	**PH-p**
Age	$\max(0{,}35 - age)$	-0.029	0.034	<0.001	0.069	-0.039	0.034	<0.001	0.117
	$\max(0, age - 35)$	0.083	0.019		0.925	0.088	0.019		0.868
Gender	female	Reference				Reference			
	male	0.856	0.304	0.003	0.481	0.900	0.306	0.002	0.531
LDH	normal	Reference				Reference			
	Below/above std limits	0.768	0.297	0.015	0.245	0.630	0.299	0.039	0.433
Primary Treatment	ABVD≤4 cycles (no RT)	Reference				Reference			
	ABVD>4 cycles (no RT)	-	-	-	-	-1.110	0.467	<0.001	0.941
	$\text{ABVD} + \text{RT}_{\le 24\text{ months}}$	-	-		-	-3.396	0.795		0.054
	$\text{ABVD} + \text{RT}_{>24\text{ months}}$	-	-		-	-1.226	0.408		0.425
	other	-	-		-	-0.616	0.422		0.907
		Global PH p-value: 0.123				Global PH p-value: 0.288			

- RT: radiotherapy; PH: proportional hazards; PH-p: p-value of PH assumption test; Std: standard.
- Reported p-values are for the terms before the possible creation of time dependent coefficients.
- Time (in months) as subscript of a term indicate the effect of the term in that time frame.
- $\max(0, x - a)$ creates the linear effect of x for values greater than a (a knot). $\max(0, a - x)$ creates the linear effect for values smaller than a but since it's a decreasing term with respect to increased x, negative coefficient estimate translates to an increased risk while a positive coefficient, decreases the risk.

Table S10-2: Final prognostic and treatment-based models for overall survival in advanced stages

		Prognostic model				Treatment-based model			
**Variable**	**Category/Spline term**	**Estimate**	**Std error**	**p-value**	**PH-p**	**Estimate**	**Std error**	**p-value**	**PH-p**
Age	$\max(0{,}40 - age)$	-0.041	0.021	<0.001	0.849	-0.046	0.021	<0.001	0.677
	$\max(0, age - 40)$	0.070	0.019		0.147	0.060	0.019		0.114
Gender	female	Reference				Reference			
	male	0.550	0.256	0.022	0.757	0.546	0.257	0.024	0.663
Albumin	$\max(0{,}32 - albumin)_{\le 18\text{ months}}$	0.098	0.036	0.019	0.322	0.070	0.038	0.037	0.332

	$\max(0,32-albumin)_{>18\ \text{months}}$	0.048	0.040		0.640	0.039	0.039		0.547
	$\max(0,albumin-32)_{\leq 18\ \text{months}}$	-0.080	0.058		0.247	-0.102	0.060		0.233
	$\max(0,albumin-32)_{>18\ \text{months}}$	0.024	0.030		0.328	0.016	0.032		0.256
Leukocyte	$\max(0,10-leukocyte)_{\leq 18\ \text{months}}$	0.313	0.068	0.005	0.112	0.310	0.069	0.013	0.144
	$\max(0,10-leukocyte)_{>18\ \text{months}}$	0.050	0.069		0.856	0.028	0.070		0.860
	$\max(0,leukocyte-10)$	0.149	0.046		0.466	0.141	0.045		0.423
	$\max(0,leukocyte-20)$	-0.263	0.125		0.305	-0.229	0.123		0.262
Primary Treatment	ABVD≤6 cycles (w/wo RT)	Reference				Reference			
	$\text{ABVD} > 6\ \text{cycles (w/wo RT)}_{\leq 18\ \text{months}}$	-	-	-	-	-1.846	0.741	0.022	0.056
	$\text{ABVD} > 6\ \text{cycles (w/wo RT)}_{>18\ \text{months}}$	-	-		-	0.517	0.272		0.591
	(Escalated) BEACOPP (w/wo RT)	-	-		-	-0.880	0.344		0.519
	other	-	-		-	0.188	0.421		0.599
		Global PH p-value: 0.813				Global PH p-value: 0.580			

- w/wo: with/without; RT: radiotherapy; PH: proportional hazards; PH-p: p-value of PH assumption test; Std: standard.
- Reported p-values are for the terms before the possible creation of time dependent coefficients.
- Time (in months) as subscript of a term indicate the effect of the term in that time frame.
- $\max(0,x-a)$ creates the linear effect of x for values greater than a (a knot). $\max(0,a-x)$ creates the linear effect for values smaller than a but since it's a decreasing term with respect to increased x, negative coefficient estimate translates to an increased risk while a positive coefficient, decreases the risk.

Validation and model comparison results of these models on Danish patients diagnosed between 2000-2018 are given below:

Figure S10-1: ROC-Calibration curves of overall survival models in early stages

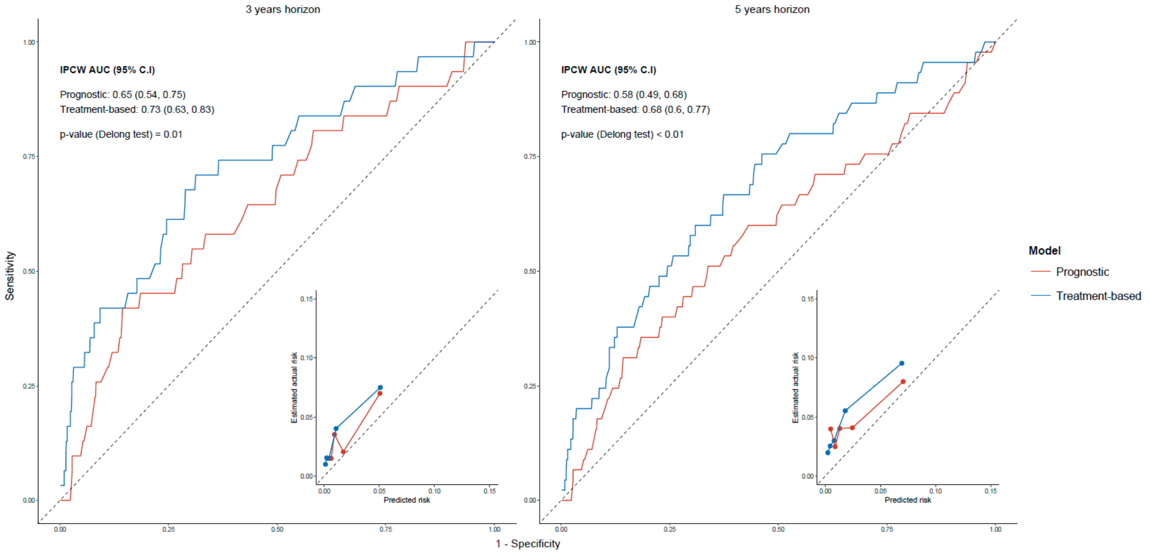

Table S10-3: Validation results of early stages models for overall survival

Model	Horizon	IPCW AUC	95% CI
Prognostic	3 years	0.648	0.545, 0.752
	5 years	0.583	0.489, 0.676
Treatment-based	3 years	0.729	0.632, 0.826
	5 years	0.683	0.601, 0.766

Table S10-4: Comparison of early stage models for overall survival based on Delong test

Horizon	Model	Reference	Delta AUC	95% CI	p-value
3 years	Treatment-based	Prognostic	0.081	0.019, 0.143	0.011
5 years	Treatment-based	Prognostic	0.100	0.051, 0.150	<0.001

Figure S10-2: ROC-Calibration curves of overall survival models in advanced stages

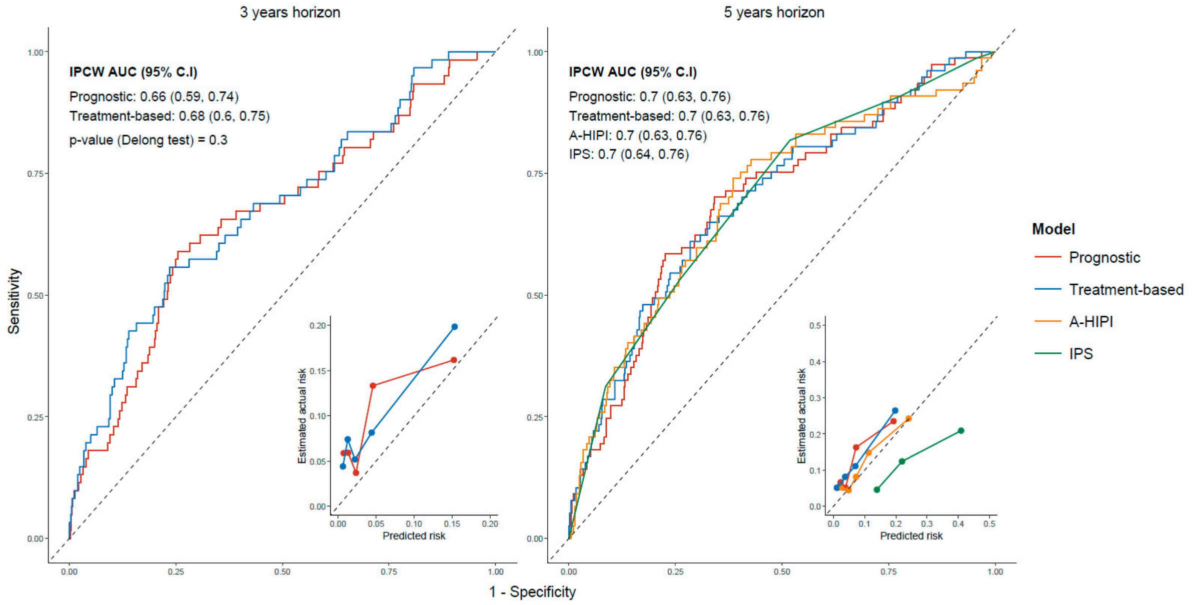

Table S10-5: Validation results of advanced stages models for overall survival

Model	Horizon	IPCW AUC	95% CI
Prognostic	3 years	0.661	0.585, 0.736
	5 years	0.695	0.631, 0.759
Treatment-based	3 years	0.676	0.602, 0.750
	5 years	0.696	0.632, 0.761
A-HIPI	5 years	0.697	0.631, 0.763
IPS	5 years	0.698	0.636, 0.760

Table S10-6: Comparison of advanced stage models for overall survival based on Delong test

Horizon	Model	Reference	Delta AUC	95% CI	p-value
3 years	Treatment-based	Prognostic	0.015	-0.014, 0.044	0.296
5 years	Treatment-based	Prognostic	0.001	-0.026, 0.029	0.925
	A-HIPI	Prognostic	0.002	-0.049, 0.053	0.935
	IPS	Prognostic	0.003	-0.053, 0.059	0.921
	A-HIPI	Treatment-based	0.001	-0.058, 0.060	0.978
	IPS	Treatment-based	0.001	-0.061, 0.064	0.963
	IPS	A-HIPI	0.001	-0.042, 0.043	0.975

Note: IPS and A-HIPI for advanced stage patients provide risk estimates at 5 years horizon so validation and comparison at 3 years horizon is not possible for them.

5 year IPCW AUC of our prognostic and treatment-based models were completely the same as both IPS and A-HIPI for overall survival. This means that the treatment effect that was detected in our data might not generalize well which may be mostly due to small number of events. To further investigate, we provide the following plots:

Figure S10-3: Effect of treatment on death from all causes through time on Dutch and Danish HL patients

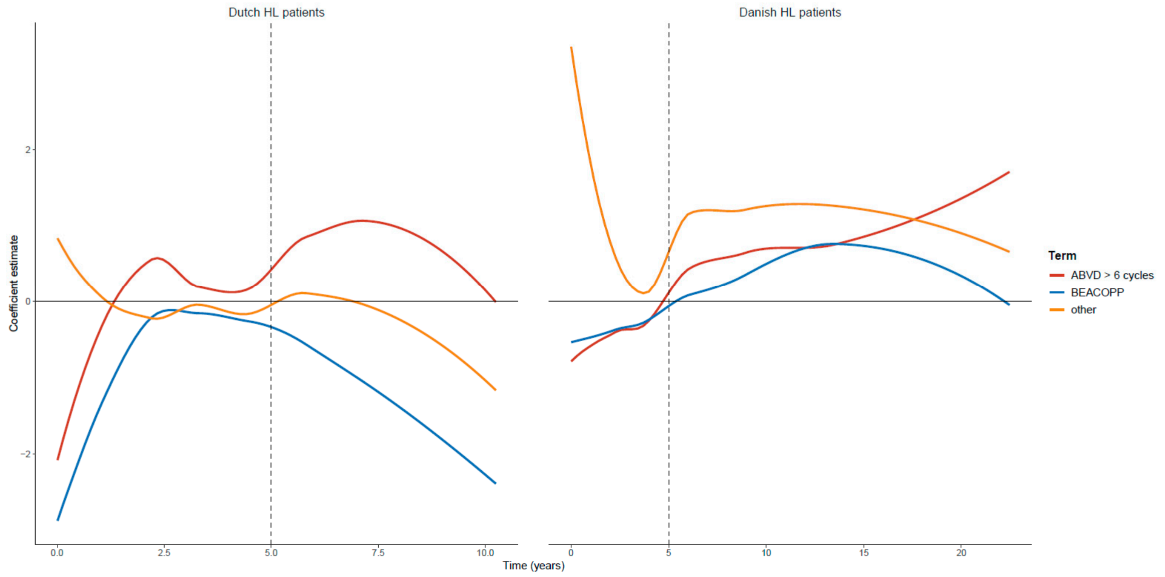

From this plot and focusing on the effects up to 5 years after diagnosis, we can see the effect of receiving >6 cycles of ABVD (with or without radiotherapy) on death (all causes) is hazardous for times after 1.5 years (left side of the plot). This is the effect that was also found by our treatment-based model for

advanced stages but we can see in Danish HL patients that the effect of receiving >6 ABVD cycles (with or without radiotherapy) on death remains mostly protective up to 5 years (right side of the plot). Effect disagreement also exists for other treatment category where it shows protective effect after 1 year in Dutch HL patients but remains hazardous at all times in Danish HL patients. These non-generalizable treatment effects in advanced stage models for overall survival explain the prevention of reaching a higher IPCW AUC by the treatment-based model against the prognostic model (without treatment).

---

[1] van Buuren, S. *Flexible Imputation of Missing Data*, 2nd ed.; Chapman & Hall/CRC: Boca Raton, FL, 2018.

[2] Therneau, T. M.; Crowson, C.; Atkinson, E. J. Using Time-Dependent Covariates and Time-Dependent Coefficients in the Cox Model. *Survival (CRAN package vignette, version 3.7-0)*, **2024**. https://CRAN.R-project.org/web/packages/survival/vignettes/timedep.pdf (accessed Aug 19, 2025).
